# Identification of novel genes associated with dysregulation of B cells in patients with primary Sjögren’s syndrome

**DOI:** 10.1186/s13075-020-02248-2

**Published:** 2020-06-22

**Authors:** Jun Inamo, Katsuya Suzuki, Masaru Takeshita, Yoshiaki Kassai, Maiko Takiguchi, Rina Kurisu, Yuumi Okuzono, Shinya Tasaki, Akihiko Yoshimura, Tsutomu Takeuchi

**Affiliations:** 1 Division of Rheumatology, Department of Internal Medicine, Keio University School of Medicine, 35 Shinanomachi, Shinjuku-ku, Tokyo, 160-8582 Japan; 2 Immunology Unit, Pharmaceutical Research Division, Takeda Pharmaceutical Company Limited, Kanagawa, Japan; 3 Integrated Technology Research Laboratories, Pharmaceutical Research Division, Takeda Pharmaceutical Company Limited, Kanagawa, Japan; 4 Rush Alzheimer’s Disease Center, Rush University Medical Center, Chicago, USA; 5 Department of Microbiology and Immunology, Keio University School of Medicine, Tokyo, Japan

**Keywords:** Primary Sjögren’s syndrome, B cells, Long non-coding RNA, Transcriptome, Gene co-expression network

## Abstract

**Background:**

The aim of this study was to identify the molecular mechanism of dysregulation of B cell subpopulations of primary Sjögren’s syndrome (pSS) at the transcriptome level.

**Methods:**

We enrolled patients with pSS (*n* = 6) and healthy controls (HCs) (*n* = 6) in the discovery cohort using microarray and pSS (*n* = 14) and HCs (*n* = 12) in the validation cohort using quantitative PCR (qPCR). Peripheral B cells acquired from these subjects were separated by cell sorting into four subsets: CD38^−^IgD^+^ (Bm1), CD38^+^IgD^+^ (naive B cells), CD38^high^IgD^+^ (pre-germinal centre B cells) and CD38^±^IgD^−^ (memory B cells). We performed differentially expressed gene (DEG) analysis and weighted gene co-expression network analysis (WGCNA).

**Results:**

Expression of the long non-coding RNA *LINC00487* was significantly upregulated in all B cell subsets, as was that of HLA and interferon (IFN) signature genes. Moreover, the normalized intensity value of *LINC00487* significantly correlated with the disease activity score of all pSS B cell subsets. Studies of human B cell lines revealed that the expression of *LINC00487* was strongly induced by IFNα. WGCNA revealed six gene clusters associated with the B cell subpopulation of pSS. Further, *SOX4* was identified as an inter-module hub gene.

**Conclusion:**

Our transcriptome analysis revealed key genes involved in the dysregulation of B cell subpopulations associated with pSS.

**Trial registration:**

Not required.

## Background

Primary Sjögren’s syndrome (pSS) is a systemic autoimmune disease characterized by exocrine gland dysfunction, which leads to dryness of the eyes and mouth [1]. Upregulation of the interferon (IFN) pathway [2] and dysregulation of B cells play a critical role in the pathogenesis of pSS. First, the loss of B cell tolerance can lead to overproduction of anti-Sjögren’s syndrome-related antigen A (anti-SSA) autoantibodies. Autoreactive B cells are important in the development of a clinical disease, because serum autoantibodies may precede the onset of dryness. Second, IFN-stimulated genes (ISGs) are upregulated in B cells in the peripheral blood as well as in the salivary gland lesions of patients with pSS [3, 4]. Third, IFNα promotes loss of tolerance and development of autoreactive B cells [5]. Genome-wide association studies identified single-nucleotide polymorphisms of ISGs that are associated with the risk of pSS [6, 7]. Further, viral infection, which enhances the IFN pathway, may contribute to the pathogenesis of pSS [8].

Non-coding RNAs, which are emerging as critical regulators of signal transduction pathways, may be involved in the immune system. Non-protein-coding microRNAs are involved in the dysregulation of B cells [9] and in the upregulation of the IFN pathway in patients with pSS [10]. Long non-coding RNAs (lncRNAs) are involved in diverse regulatory functions, including the modulation of chromatin structure and post-transcriptional regulation affecting the stability of mRNAs and proteins [11]. Recent evidence indicates that lncRNAs, which have become the focus of studies on autoimmune diseases, may contribute to the pathogenesis of pSS through multiple signal transduction pathways [12]. For example, differentially expressed lncRNAs in the labial salivary glands of pSS patients were identified using microarray analysis [13]. In the multi-omics study of whole blood specimen, the ratio of B cell subpopulation within white blood cells was associated with the expression of pSS-associated signature genes, raising the necessity of in-depth investigation including lncRNAs about the link between dysregulated B cells and transcriptome [14].

Considering that the gene expression pattern is tissue-specific [15], it is better to focus on the target cell subpopulation to avoid noise from other cells in transcriptome analysis. However, we are unaware of studies that focus on the effects of dysregulation of transcriptomes, including lncRNAs, of B cells on the pathogenesis of pSS. Serum level of B cell-activating factor was associated with disease activity of pSS [16–18]. In addition, the distribution of peripheral B cell subsets is profoundly altered in patients with pSS [19]. Staining for IgD/CD38 is helpful for studying circulating B cell subsets, separating developmental stages from naive to memory B cells (Bm1 to Bm5) [20]. Using this gating strategy, we previously reported that the proportion of pre-GC B cells (IgD^+^/CD38^high^) was higher in patients with pSS compared with healthy controls and associated with clinical traits, such as disease activity [19], indicating the importance of evaluating the involvement of each B cell subset in the pathogenesis of pSS.

To determine the role of the B cell subset in the pathogenesis of pSS, we investigated potentially significant genes according to their differential levels of expression and connectivity with other genes. Thus, the current study consists of two sections: differentially expressed gene (DEG) analysis and weighted gene co-expression network analysis (WGCNA). First, we describe the upregulation of the interferon signalling pathway and the differential expression of genes encoding human leucocyte antigen (HLA) molecules and *LINC00487* in B cell subpopulations of patients with pSS compared with healthy controls (HCs). The expression levels of *LINC00487* correlated with the disease activity of pSS and IFN signature genes, and *LINC00487* was induced by IFNα. Second, using WGCNA, we identified genes of co-expression networks specific to a B cell subset of patients with pSS, suggesting that aberrant molecular interactions in B cells contribute to the aetiology of pSS.

## Methods

### Patients and controls

**Fig. 1:**
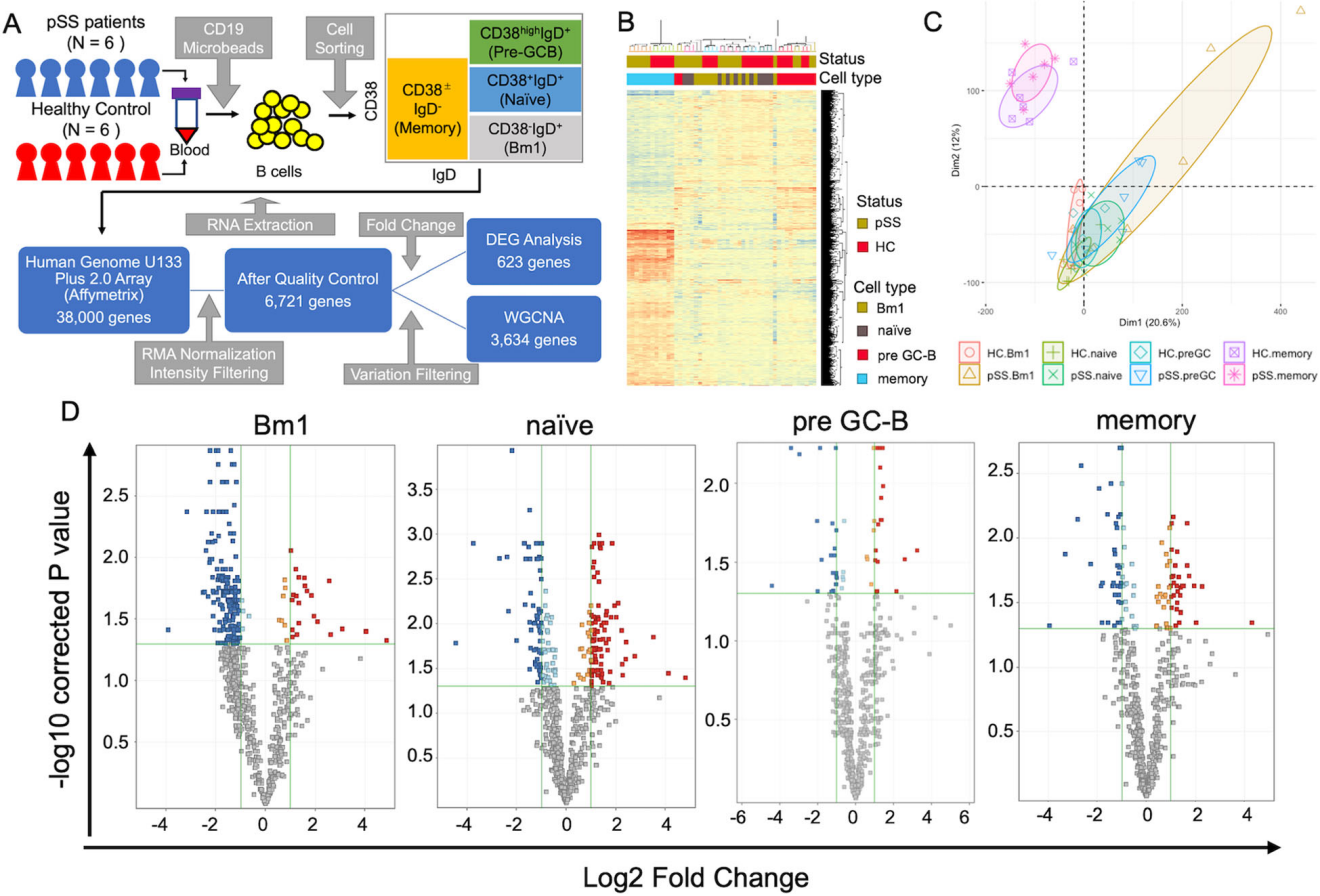
Gene expression profile of B cell subsets in patients with pSS and HCs. **a** Study protocol. **b** Hierarchical clustering analysis and **c** principal component analysis to summarize the dissimilarity of the transcriptome of each B cell subset. Rows correspond to genes and columns to samples. The ellipse shows the 50% confidence interval of the value of principal component analysis. **d** Dysregulated genes are shown in the volcano plot. Horizontal green lines show the cut-off of the *p* value indicating a significant difference, and the vertical green lines show a log2-fold change. DEG, differentially expressed gene; GC-B, germinal centre B cell: HC, healthy control; pSS, primary Sjögren’s syndrome; WGCNA, weighted gene co-expression network analysis

The study protocol is shown in Fig. 1a. We enrolled patients with pSS (*n* = 6) and HCs (*n* = 6) matched for age and sex (Supplementary Table 1). The patients with pSS fulfilled the 2002 American-European criteria for SS [21] and the 2012 American College of Rheumatology classification criteria for SS [22]. All patients were female and had a symptom of dryness, anti-SSA antibodies and biopsy-proven sialadenitis. The patients had not received immunosuppressive therapy, and the HCs did not have immunological disorders. Clinical and serological information of patients with pSS were collected from their medical records. The disease activity of pSS was assessed according to the guidelines of the European League against Rheumatism and the Primary Sjögren’s Syndrome Disease Activity Index (ESSDAI) [23, 24]. The distribution of ESSDAI domain and clinical information of individual patients were described in Supplementary Table 17 and Supplementary Table 18.

This study was performed in accordance with relevant guidelines and regulations. The Ethics Committee of Keio University School of Medicine approved this study (IRB No. 20110258), and written informed consent was obtained from each subject before blood collection.

### Cell sorting

Peripheral blood mononuclear cells from patients with pSS and HCs were separated using gradient centrifugation with Lymphoprep (Axis-Shield; Oslo, Norway). Gating strategy was shown in Supplementary Figure 1. Peripheral CD19^+^ B cells were prepared with anti-CD19 antibody-coated microbeads (Miltenyi Biotec). As previously reported [19], the peripheral CD19^+^ B cells were incubated with anti-IgD and CD38 antibodies for fluorescence-activated cell sorting (FACS) analysis (FACSAria III flow cytometer, BD Biosciences). We defined subsets of B cells as follows: Bm1 cells, CD38^−^IgD^+^; naive B cells, CD38^+^IgD^+^; pre-germinal centre (pre-GC) B cells, CD38^high^IgD^+^; and memory B cells, CD38^±^IgD^−^.

### DEG analysis

Total RNA was extracted from B cell subsets and transcribed into cDNA using NucleoSpin RNA (Macherey Nagel) and ReverTra Ace qPCR RT Master Mix (Toyobo). Gene expression was measured using the Human Genome U133 Plus 2.0 Array (Affymetrix). We applied percentile shift normalization to the raw signal data acquired from a microarray and annotated each probe with its gene symbol using the GeneSpring software (Agilent Technologies). Probes with interquartile ranges in the lowest 20% were excluded. We next selected probes with > 2.0 changes for pSS vs HCs in any one B cell subset to identify DEGs. We controlled for the false discovery rate using the Bonferroni multiple testing-corrected *p* value < 0.05. To functionally characterize DEGs identified in each B cell subpopulation from microarray analysis, we performed a pathway analysis using Enrichr’s plugin [25] BioPlanet [26]. The BioPlanet database incorporates more than 1500 human pathways sourced from publicly available, manually curated sources. In a pathway analysis, *p* value was adjusted using the Benjamini-Hochberg method for correction for multiple hypotheses testing.

### WGCNA

To explore novel gene co-expression networks and common hub genes, we created another gene set. In order to select genes that are steadily expressed in each B cell subpopulation of pSS and HCs (totally, 8 subpopulations; Bm1, naive, pre-GC and memory B cells of pSS, and Bm1, naive, pre-GC and memory B cells of HCs), we removed genes with coefficients of variation greater than 0.3 according to each subpopulation after intensity filtering and applied them to the WGCNA R package [27] following the package’s tutorial. Briefly, we applied step-by-step construction of the gene network and identification of modules to fileted gene expression dataset. First, we chose the proper soft-thresholding power based on the criterion of approximate scale-free topology. After calculating the adjacencies with selected soft-thresholding power, we transformed the adjacency into a topological overlap matrix and calculate the corresponding dissimilarity. Then, we used hierarchical clustering to produce a hierarchical clustering tree of genes. Module identification amounts to the identification of individual branches; we cut the branches off the dendrogram with setting minimum module size 30. As a result, 6 and 5 modules were generated in B cells of patients with pSS and HCs, respectively. Further, to annotate and estimate the regulatory mechanism of a co-expressed gene network, we investigated the pathways, upstream regulators and functions associated with modules by using Ingenuity Pathway Analysis (IPA).

### Cell culture and real-time quantitative PCR

B cell lines (Ramos, CCRF, U266B1) (each 1.0 × 10^5^/mL) in RPMI 1640 medium (American Type Culture Collection) supplemented with 10% foetal calf serum were treated with different concentrations of IFNα, IFNγ and TNFα for 48 h. We next isolated total RNA that was transcribed into cDNAs using NucleoSpin RNA (Macherey Nagel) and ReverTra Ace qPCR RT Master Mix (Toyobo). qPCR was performed using PowerUp SYBR Green Master Mix (Thermo Fisher Scientific). The levels of total cellular RNA were similar amongst the cell lines. At least two biological repeats were performed for each experiment. The levels of *GAPDH* mRNA were used to adjust the values of target transcripts determined using qPCR analyses. We validated gene expression levels in CD19^+^ B cells of patients with pSS (*n* = 14) and HCs (*n* = 12) (Supplementary Table 2) using quantitative PCR (qPCR) analysis. The sequence of primers for qPCR analysis was described in Supplementary Table 3.

### Statistics

Continuous values are shown as the median and range of values. Differences between the groups were analysed using the Mann-Whitney test for continuous variables. Correlations were analysed using Spearman’s correlation coefficient, unless otherwise noted. *p* < 0.05 indicates a significant difference. Statistical tests were conducted using R software version 3.5.2.

## Results

### Identification of gene signatures in B cell subsets of pSS

First, we visualized the gene expression profile after intensity filtration. Using principal component and hierarchical clustering analyses, we found that clinical status, namely pSS or HCs, had less influence on phenotype than the type of B cell subset (Fig. 1b, c). Memory B cells, in particular, had a distinct expression pattern compared with those of other cell types.

To determine the gene signatures of B cell subset of patients with pSS, we identified DEGs and performed pathway analyses. Gene selection yielded 623 significant genes (Supplementary Table 4). Analysis of changes in the expression indicated that 23, 92, 18 and 32 genes were upregulated in Bm1, naive, pre-GC and memory B cell subsets, respectively (Fig. 1d).

**Fig. 2:**
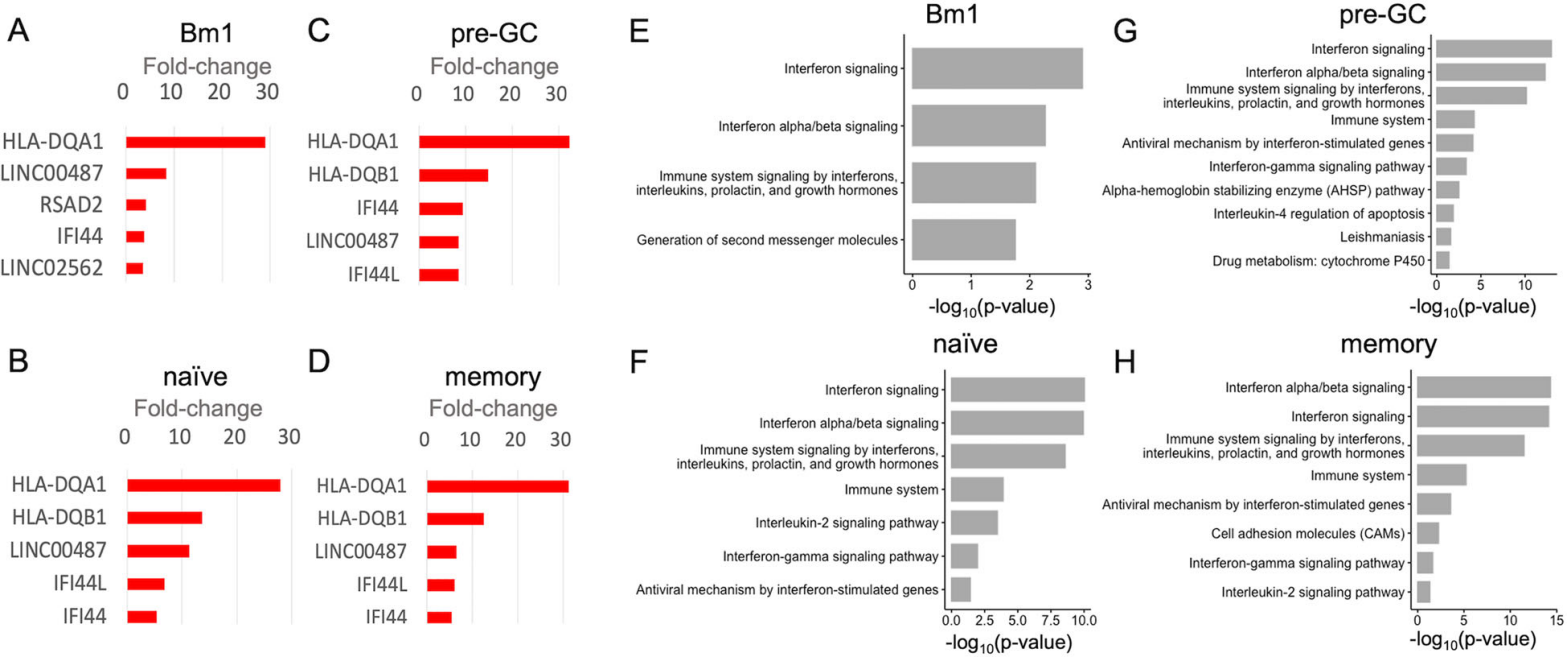
Gene signatures of B cell subsets of patients with primary Sjögren’s syndrome. **a**–**d** The top upregulated five genes in Bm1 (**a**), naive (**b**), pre-GC (**c**) and memory (**d**) B cell subset of patients with pSS compared with those of the HCs. **e**–**h** Upregulated pathways in Bm1 (**e**), naive (**f**), pre-GC (**g**) and memory (**h**) B cell subset of patients with pSS compared with those of the HCs. GC, germinal centre

Consistent with previous reports, ISGs were upregulated in B cells of pSS compared with those of HCs (Fig. 2a–d). Further, HLA genes were similarly upregulated. Pathway analysis demonstrated that the interferon pathway was the most significantly upregulated pathway in all of B cell subsets (Fig. 2e–h). In contrast, regarding downregulated genes, there was no enriched pathway. We found that a lncRNA, *LINC00487*, was amongst the 15 probes significantly upregulated in all B cell subsets of pSS compared with those of HCs (Supplementary Table 5). The fold changes of *LINC00487* expression were as follows: Bm1, 8.4 (*p* = 0.038); naive, 11.3 (*p* = 0.014); pre-GC, 8.5 (*p* = 0.089); and memory, 6.37 (*p* = 0.078) (Fig. 2a–d). There were no significant differences amongst the B cell subsets examined for HCs or pSS patients (Supplementary Figure 2).

### Expression of LINC00487 correlates with IFN signature genes and disease activity score of pSS

**Fig. 3:**
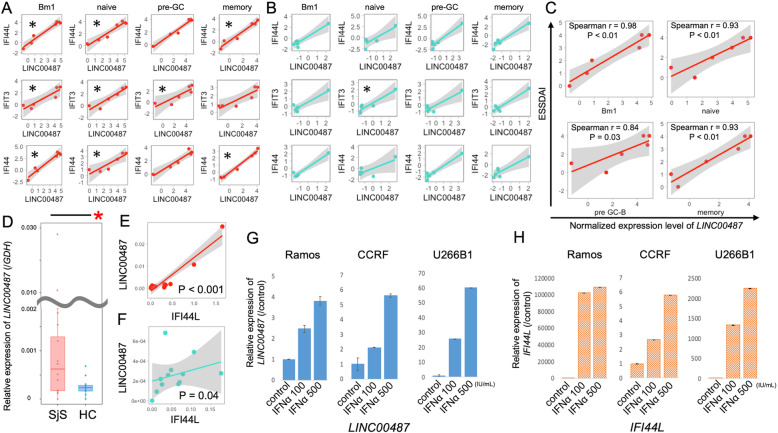
Characteristics of *LINC00487*. **a**, **b** Correlation between *LINC00487* and interferon signature genes (*IFI44L*, *IFIT3*, *IFI44*) of pSS (**a**) and HCs (**b**) according to the subset. Each row corresponds to an interferon signature gene and columns to a B cell subset. Significant *p* value was shown in each plot. *p < 0.05; Spearman’s correlation test. **c** Scatter plot of disease activity scores and normalized expression levels of *LINC00487* in Bm1, naive, pre-GC and memory subset of patients with pSS. **d**–**f** qPCR analysis of *LINC00487* in primary human CD19^+^ B cells. Comparison of the relative expression of *LINC00487* (/*GDH*) between patients with pSS and HCs (**d**) (*p < 0.05; the Mann-Whitney test), and correlation plot of relative expression of the *LINC00487* (/*GDH*) and interferon-*induced protein 44-like* gene (/*GDH*) in patients with pSS (**e**) and HCs (**f**). **g**, **h** qPCR analysis of B cell lines after treatment with IFNα. B cell lines were treated for 48 h. Results are represented as the mean ± standard deviation. ESSDAI, EULAR Sjögren’s Syndrome Disease Activity Index; GC-B, germinal centre B cell; GDH, glyceraldehyde 3-phosphate dehydrogenase; HC, healthy control; IFI44, interferon-induced protein 44; IFI44L, interferon-induced protein 44 like; IFIT3, interferon-induced protein with tetratricopeptide repeats 3; pSS, primary Sjögren’s syndrome

To determine the characteristics of *LINC00487*, we identified the top three genes that correlated with *LINC00487* amongst all of the subsets (*interferon-induced protein 44 like* [*IFI44L*], *interferon-induced protein with tetratricopeptide repeats 3* [*IFIT3*] and *interferon-induced protein 44* [*IFI44*], in the order of low *p* value). All three genes were known ISGs. Expression of *LINC00487* and these ISGs were significantly correlated in more subsets of pSS compared with HCs (9 vs 1 subset, respectively) (Fig. 3a, b). Further, the clinical disease activity score was correlated with the expression level of *LINC00487* in all pSS B cell subsets: Bm1, *r* = 0.98 (*p* < 0.01); naive, *r* = 0.93 (*p* < 0.01); pre-GC, *r* = 0.84 (*p* = 0.03); and memory, *r* = 0.93 (*p* < 0.01) (Fig. 3c). However, it should be noted that only patients with low ESSDAI scores (< 5) were included in the current cohort.

Using a validation cohort, we tried to conduct a qPCR analysis using each B cell subset. However, because *LINC00487* was expressed at a very low level in HCs, we could not sort enough each B cell subset from them to detect the expression of *LINC00487*. Therefore, considering that upregulation of *LINC00487* was common in all B cell subsets in microarray cohort, we used a bulk of CD19^+^ B cells in the validation cohort. As a result, the expression of *LINC00487* in CD19^+^ B cells of patients with pSS was significantly upregulated compared with HCs (*p* = 0.035) (Fig. 3d). The same as Fig. 3a and b, the expression of *LINC00487* was correlated with *IFI44L* with lower *p* value in patients with pSS (*p* < 0.001) (Fig. 3e) compared with that of HCs (*p* = 0.04) (Fig. 3f). To confirm the induction of *LINC00487* in response to IFNα, we used three types of B cell lines and measured the *LINC00487* expression using qPCR. As with *IFI44L*, *LINC00487* was strongly induced by treatment with IFNα (Fig. 3e, f), unlike TNFα and IFNγ (Supplementary Figure 3A), suggesting that IFNα is an upstream regulator of *LINC00487*. Compared with clinical features, the expression of *LINC00487* tended to be higher in patients with higher serum IgG levels and lower lymphocyte counts which were characteristic for pSS (Supplementary Figure 3B).

### Co-expression gene networks constructed for each B cell subset

To identify transcriptional networks in the B cells of patients with pSS, we performed WGCNA. WGCNA can identify novel gene interactions which might be missed in the DEG analysis, because WGCNA is based on correlations of gene expression [25].

**Fig. 4:**
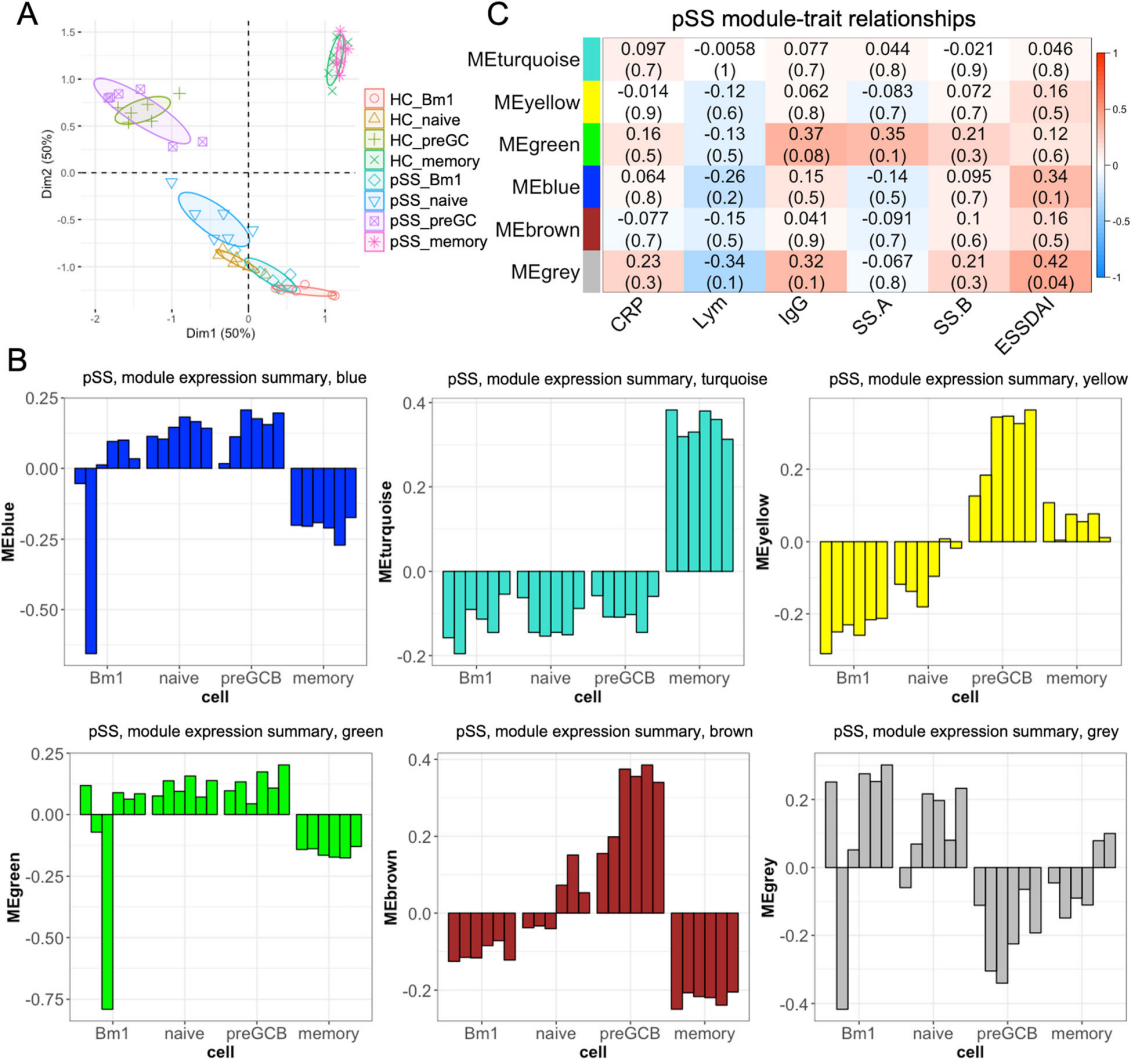
Weighted gene co-expression network analysis of B cell subsets of patients with pSS. **a** Principal component analysis using the gene set produced is generated using WGCNA. The ellipse shows the 50% confidence interval of the value of principal component analysis. **b** Module eigengene of each B cell subset of patients with pSS. The *y*-axis displays the values of the module eigengene, and the *x*-axis displays each cell subset. **c** The heatmap shows module-trait associations. Each row corresponds to a module’s first eigengene and columns to a measured value of trait. Each cell contains the corresponding Spearman’s correlation coefficient (*p* value) between a measured value of each trait and a module’s first eigengene. CRP, C-reactive protein; ESSDAI, EULAR Sjögren’s Syndrome Disease Activity Index; GC-B, germinal centre B cell; Lym, lymphocyte count; SS.A, anti-Sjögren’s syndrome-related antigen A; ME, module eigengene; SS.B, anti-Sjögren’s syndrome-related antigen B

After selection, we identified a new set comprising 3634 genes (Supplementary Tables 6–7). In the principal component analysis using these gene sets, Bm1 and naive B cells in pSS tended to be closer to the expression patterns of a more highly differentiated subset compared to those in HCs, i.e. Bm1 to naive and naive to pre-GC (Fig. 4a). WGCNA identified 6 modules in pSS and an association between cell subsets and modules (Fig. 4b). For example, the yellow and brown modules are associated with the pre-GC B cell subset, whereas the turquoise module is associated with the memory B cell subset. The grey module of pSS was enriched in Bm1/naive B cell subsets with various ranges of eigengenes. In the analysis to detect an association between modules and clinical traits, the grey module significantly correlated with the clinical disease activity score (Fig. 4c). Similarly, another five modules were identified in HCs (Supplementary Figure 4A). The module pairs with the greatest number of genes in each pSS module overlapping with either HC modules were blue (pSS)–turquoise (HC), brown (pSS)–brown (HC), grey (pSS)–blue (HC), green (pSS)–turquoise (HC), yellow (pSS)–blue (HC) and turquoise (pSS)–turquoise (HC) (Supplementary Figure 5). All of the above pairs overlapped more than half of the gene list, suggesting that the overall picture of the B cell modules in pSS and the B cell modules in HCs was similar.

**Fig. 5:**
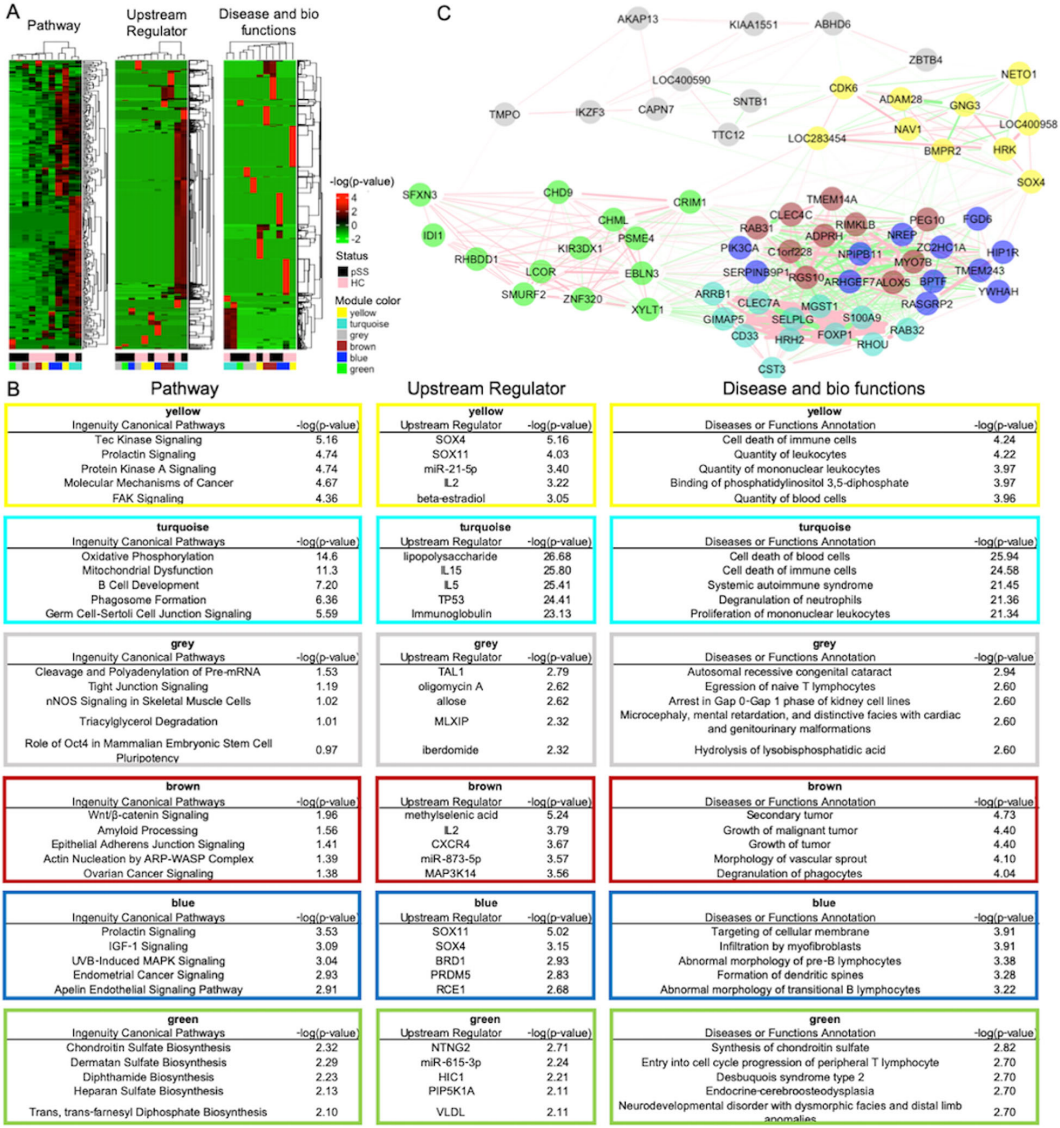
Gene network characteristics and hub genes in the gene networks of B cells of pSS. **a** Hierarchical clustering analysis with a heatmap of enriched pathways (left), upstream regulators (middle) and disease/functions (right). **b** Top five significantly enriched canonical pathways (left), upstream regulators (middle) and disease and biological functions (right) in the modules of pSS. The colour of each frame corresponds to a module colour. **c** Associations of intra- and inter-modular hub genes in patients with pSS. Node colour corresponds to module colour. The pink-coded edge represents the correlation, and the green-coded edge represents an inverse correlation. The width of the edge reflects the absolute weight of a correlation

To extract the characteristics of pSS modules, we performed a functional analysis using Ingenuity Pathway Analysis (IPA). The cluster of enriched pathway, upstream regulator and disease/biological functions in modules of pSS were distinguished from those of HCs (Fig. 5a and Supplementary Tables 8–13). The top five most significantly enriched pathways, regulators and disease/biological functions in modules of pSS are shown in Fig. 5b. In the upstream regulator analysis, transcriptional regulators such as Sry-related high-mobility group box (Sox) family such as *SOX4* were identified in the yellow and blue modules of pSS. Focusing on specificity (Supplementary Figure 6 and Supplementary Tables 14–16), mir-21-5p was identified as the top significant regulator in the yellow module of pSS. To search for hub genes, we described a co-expressed gene network using top highly interconnected genes defined by a topological overlap in each module (Fig. 5c). Several genes bridged inter-modules, namely hub genes, such as *SOX4*. *SOX4* was not identified in the co-expression network of HCs (Supplementary Figure 4B), suggesting its involvement in the dysregulation of B cells in pSS.

## Discussion

In the present study, we conducted a transcriptome analysis of B cell subpopulations. First, we found that the expression of *LINC00487* was upregulated in B cell subsets derived from patients with pSS. Further, *LINC00487* expression significantly correlated with disease activity and was induced by IFNα stimulation. Second, using WGCNA, we identified several key networks and hub genes in the B cells of patients with pSS.

IFN signalling is a central component of the pathogenesis of pSS [28]. Type 1 IFN signalling plays a crucial role in the development of autoreactive B cells in a mouse model of autoimmune disease [5]. Principal component analysis using gene sets for WGCNA demonstrated that Bm1/naive B cells of pSS were clearly distinguished from those of HCs and showed a trend of expression patterns closer to the more highly differentiated subset. These results suggest that molecular dysfunction, such as disruption of peripheral tolerance, might start at an early stage in the maturation of B cells. Indeed, the frequencies of naive B cells expressing autoreactive antibodies are significantly increased in patients with pSS [29].

Interestingly, expression of the HLA class II gene was upregulated. GWAS studies of pSS found associations of *HLA-DQA1* and *HLA-DQB1* loci [6, 7]. Further, IFNα induces the expression of *HLA-DQA1* [30]. In patients with pSS, *HLA-DQA1* and *HLA-DQB1* alleles are associated with higher concentrations of anti-SSA and SSB antibodies [31]. These findings suggest that aberrant interactions amongst IFN signalling and HLA class II genes may trigger the breakdown of B cell tolerance, leading to the development of pSS.

*LINC00487* was upregulated in all B cell subsets of pSS, and its expression significantly correlated with the disease activity scores of pSS and ISGs, although the ESSDAI score of our patients skewed toward the low end. Moreover, we found that IFNα was an upstream regulator of *LINC00487* in B cells. The sequence of *LINC00487*, which belongs to the class of long intergenic non-coding RNAs and resides on human chromosome 2, is atypically long (> 40,000 bases). Although *LINC00487* is one of the hub genes in the normal development of human B cells [32], many of its properties, including its function, are unexplained. Further, there is no ortholog or paralog of this gene in species other than humans that may provide clues to its function in human cells. However, according to AceView, one of three transcriptional variants derived from *LINC00487* has the potential to encode a protein in silico prediction [33].

Four genomic locations are considered candidate enhancers of *LINC00487* transcription. The targets of the regulators of *LINC00487* overlap with those of other ISGs [34]. Moreover, referring to the public transcriptome database of microarray analyses of healthy humans, expression of *LINC00487* is higher specifically in centroblasts and centrocytes of the germinal centre [35]. IFNα promotes the autoreactivity of B cells via germinal centre pathways [5]. Further, *LINC00487* expression is upregulated in the subgroup of diffuse large B cell lymphoma with molecular characteristics of germinal centre B cells, compared with other subgroups, and is associated with the efficacy of B cell depletion therapy [36]. Therefore, our study suggests that upregulation of *LINC00487* expression in all B cell subsets may reflect or regulate the enrichment of a germinal centre-like reaction by IFNα from an early stage of B cell development, leading to B cell autoreactivity in patients with pSS.

Gene co-expression analysis revealed an aberrant network in B cell subpopulations. The top significant upstream regulator of the grey module of pSS, which was associated with clinical disease activity score and enriched in the early stage of B cell development in pSS, was the gene encoding T cell acute lymphocytic leukaemia protein 1 (*TAL1*). B cell development is stringently controlled by stage-specific transcription factors. The transcription factor *TAL1* regulates genes such as IKAROS family zinc finger 3 (*IKZF3*), which is one of the hub genes in the grey module of pSS (Fig. 5c). *IKZF3* is a lineage-specific transcription factor that is important in the regulation of B cell proliferation and development [37].

In the pre-GC B cell-associated module of pSS, *SOX4* was identified as an upstream regulator and a hub gene. In mice, *SOX4* regulates the differentiation of early-stage B cells by activating the expression of *Rag1* and *Rag2* [38]. Further, *SOX4* contributes to the formation of ectopic lymphoid-like structures via promoting CXCL13, which is a ligand of CXCR5 on naive B cells and critical for migration into the light zone of germinal centre undergoing somatic hypermutation, production from PD-1^hi^CXCR5^−^CD4^+^ T cells [39]. Additionally, in proteome analysis, CXCL13 positively correlates with the disease activity score and serum IgG levels of patients with pSS [19]. Further study is needed to explore the role of *SOX4* in mature B cells.

Further, we identified miR-21 as an upstream regulator of the yellow module of pSS. Although miR-21 is upregulated in peripheral blood mononuclear cells of pSS [40], the present study is the first to propose its involvement in pSS via B cell dysregulation. Interestingly, miR-21 regulates the immune response of memory T cells via induction of transcription networks, such as *SOX4* [41], implying that the interaction between miR-21 and *SOX4* may also affect the function of B cells.

pSS is an enormously heterogeneous disease from the molecular point of view. In WGCNA, despite filtering out genes with high variation in each cell subpopulation, module expression showed the strong variation amongst samples in the same group, in particular Bm1 subset (Fig. 4b). One of the reasons for the high variability seen in Bm1 subset may be related to the fact that some cells with the CD38^−^IgD^+^ express CD27 [20]. Namely, the CD38^−^IgD^+^ B cell subpopulation includes both naive Bm1 cells and IgD^+^ memory B cells. However, the finding in the current study suggested that there might be different regulatory mechanisms within each subgroup, although it was information that could not be analysed due to the limited sample size.

The current study suffers from several limitations. First, patients in our cohort have low disease activity/severity. Because there were few patients with high disease activity before immunosuppressive treatment, it was difficult to include such patients in this study. Therefore, the correlation between the expression of *LINC00487* and disease activity score is needed to be validated in another cohort including patients with high ESSDAI scores. Second, healthy controls were younger than the pSS patients, and this could be a confounding variable. Third, the sample size was limited. To overcome the limitation about the small sample size, we validated by qPCR using another cohort, supporting the results derived from microarray analysis. However, regarding WGCNA, we could not include subjects enough to replicate by qPCR.

## Conclusions

Our focus on B cell subpopulation using a multi-level approach employing the analysis of DEGs and WGCNA identified significant genes and networks as novel players in the pathogenesis of pSS. To confirm our results, further study is needed.

## Supplementary information

**Additional file 1 **: **Table S1**. Characteristics of patients with primary Sjögren’s syndrome (pSS) and healthy controls (HCs) in microarray analysis cohort. **Table S2**. Characteristics of patients with primary Sjögren’s syndrome (pSS) and healthy controls (HCs) in validation cohort. **Table S3**. Sequence of primers for qPCR analysis. **Table S4**. List of annotated probes that were significantly (*p* < 0.05) upregulated with ≥2-fold changes in any B cell subset of pSS compared with those of HCs. **Table S5**. List of probes that were significantly (*p* < 0.05) upregulated with ≥2-fold changes in all B cell subpopulations compared with those of HCs. **Tables S6 and S7**. List of probes in gene co-expression modules of pSS (Table 6) and HCs (Table 7). To quantify associations of individual genes with the disease activity score (EULAR Sjögren’s Syndrome Disease Activity Index, ESSDAI), we defined Gene Significance (GS) as the absolute value of the correlation between each gene and ESSDAI. P.GS represents the *p*-value of GS. For each module, we defined a quantitative measure of module membership (MM) as the correlation between the module eigengene and the gene expression profile. P.MM means the *p* value of MM. This allowed us to quantify the similarities among all genes of every module. **Table S8**. List of canonical pathways associated with gene co-expression modules of pSS. **Table S9**. List of upstream regulators associated with gene co-expression modules of pSS. **Table S10**. List of disease and functions associated with gene co-expression modules of pSS. **Table S11**. List of canonical pathways associated with the identified gene co-expression modules of HCs. **Table S12**. List of upstream regulators associated with gene co-expression modules of HCs. **Table S13**. List of disease and functions associated with gene co-expression modules of HCs. **Table S14**. List of canonical pathways specific to pSS. **Table S15**. List of upstream regulators specific to pSS. **Table S16**. List of disease and functions specific to pSS. **Table S17**. The distribution of ESSDAI of patients with pSS. **Table S18**. The detailed clinical information of patients with pSS. **Fig. S1.** Gating strategy The gating strategy is shown. To evaluate CD19+ B cells along two axes, CD19+ B cells were first divided from peripheral blood mononuclear cells (A). Then, we defined subsets of B cells as follows: Bm1 cells; CD38-IgD+, naïve B cells; CD38 + IgD+, pre-germinal centre (pre-GC) B cells; CD38highIgD+ and memory B cells; CD38 ± IgD- (B). **Fig. S2**. Relative expression levels of *LINC00487* in B cell subsets. GCB, germinal centre B cell: HC, healthy controls: pSS, primary Sjögren’s syndrome. **Fig. S3**. Characteristics of *LINC00487*. **Fig. S4**. Weighted gene co-expression network analysis of B cell subsets of HCs. **Fig. S5**. Venn diagram of genes in module. **Fig. S6**. Top five significantly enriched canonical pathways (left), upstream regulators (middle) and disease/functions (right) specific for pSS. The colour of each frame corresponds to module-colour.

## Data Availability

The transcriptome data are available at the GEO database. The accession code is GSE135809. All custom computer codes in the generation or processing of the described data are available upon reasonable request. Supplementary Tables 4–18 and R script for WGCNA are deposited in figshare (https://figshare.com/articles/Supplementary_Table_4-14/11683959).

## Abbreviations

ESSDAI: EULAR Sjögren’s Syndrome Disease Activity Index
FACS: Fluorescence-activated cell sorting
GC-B: Germinal centre B cell
HC: Healthy control
HLA: Human leucocyte antigen
IFN: Interferon
IKZF3: IKAROS family zinc finger 3
ISGs: Interferon-stimulated genes
lncRNA: Long non-coding RNA
pSS: Primary Sjögren’s syndrome
TAL1: T cell acute lymphocytic leukaemia protein 1
WGNCA: Weighted gene co-expression network analysis
