# Supplementary material for: Identification of novel genes associated with dysregulation of B cells in patients with primary Sjögren’s syndrome
Source: Arthritis Res Ther. 2020 Jun 22;22:153. doi: 10.1186/s13075-020-02248-2 (PMC7310138; doi:10.1186/s13075-020-02248-2)
Supplement: Supplementary file 1 — Additional file 1 : Table S1. Characteristics of patients with primary Sjögren’s syndrome (pSS) and healthy controls (HCs) in microarray analysis cohort. Table S2. Characteristics of patients with primary Sjögren’s syndrome (pSS) and healthy controls (HCs) in validation cohort. Table S3. Sequence of primers for qPCR analysis. Table S4. List of annotated probes that were significantly (p < 0.05) upregulated with ≥2-fold changes in any B cell subset of pSS compared with those of HCs. Table S5. List of probes that were significantly (p < 0.05) upregulated with ≥2-fold changes in all B cell subpopulations compared with those of HCs. Tables S6 and S7. List of probes in gene co-expression modules of pSS (Table 6) and HCs (Table 7). To quantify associations of individual genes with the disease activity score (EULAR Sjögren’s Syndrome Disease Activity Index, ESSDAI), we defined Gene Significance (GS) as the absolute value of the correlation between each gene and ESSDAI. P.GS represents the p-value of GS. For each module, we defined a quantitative measure of module membership (MM) as the correlation between the module eigengene and the gene expression profile. P.MM means the p value of MM. This allowed us to quantify the similarities among all genes of every module. Table S8. List of canonical pathways associated with gene co-expression modules of pSS. Table S9. List of upstream regulators associated with gene co-expression modules of pSS. Table S10. List of disease and functions associated with gene co-expression modules of pSS. Table S11. List of canonical pathways associated with the identified gene co-expression modules of HCs. Table S12. List of upstream regulators associated with gene co-expression modules of HCs. Table S13. List of disease and functions associated with gene co-expression modules of HCs. Table S14. List of canonical pathways specific to pSS. Table S15. List of upstream regulators specific to pSS. Table S16. List of disease and functions specific to pSS [file 13075_2020_2248_MOESM1_ESM.pdf]

Identification of novel genes associated with dysregulation of B cells in patients with primary Sjögren's syndrome

Jun Inamo[1], Katsuya Suzuki[1], Masaru Takeshita[1], Yoshiaki Kassai[2], Maiko Takiguchi[2], Rina Kurisu[2], Yuumi Okuzono[2], Shinya Tasaki[3,4], Akihiko Yoshimura[5], Tsutomu Takeuchi[1]

Supplementary Table 1. Characteristics of patients with primary Sjögren's syndrome and healthy controls in microarray analysis cohort.

Characteristic	pSS<br>N=6	HC<br>N=6
Age, years	61 (41-73)	41 (26-56)
Female, %	100	100
Disease duration, years	2.5 (1-4)	.
Ocular symptoms, %	17	.
Oral symptoms, %	100	.
Anti-SSA positivity, %	100	.
Anti-SSB positivity, %	83	.
IgG, mg/dL	1762<br>(1321-2275)	.
IgA, mg/dL	255<br>(163-524)	.
IgM, mg/dL	74 (56-141)	.
Lymphocytic sialadenitis with focus score ≥1	100	.
ESSDAI	3 (0-4)	.
ESSDAI ≥1, %	83	

Median and range of values are shown.

ESSDAI, EULAR Sjögren's Syndrome Disease Activity Index: HC, healthy control: pSS, primary Sjögren's syndrome: SSA, Sjögren’s syndrome-related antigen A: SSB, anti-Sjögren’s syndrome-related antigen B

Supplementary Table 2. Characteristics of patients with primary Sjögren's syndrome and healthy controls in validation cohort.

Characteristics	pSS N=14	HC N=12
Age, years	62 (31-85)	35 (29-43)
Female, %	92%	35%
Disease duration, years	3.0 (0.2-20)	.
Ocular symptoms, %	57%	.
Oral symptoms, %	71%	.
Anti-SSA positivity, %	85%	.
Anti-SSB positivity, %	50%	.
IgG, mg/dL	1373 (979-2474)	.
IgA, mg/dL	277 (185-447)	.
IgM, mg/dL	88.5 (53-160)	.
Lymphocytic sialadenitis with focus score ≥1	28%	.
ESSDAI	2 (0-8)	.

Median and range of values are shown.

ESSDAI, EULAR Sjögren's Syndrome Disease Activity Index: HC, healthy control: pSS, primary Sjögren's syndrome: SSA, Sjögren’s syndrome-related antigen A: SSB, anti-Sjögren’s syndrome-related antigen B

Supplementary Table 3. Primers used in this study.

Gene name	Orientation	Sequence of the primer (5' to 3')
LINC00487	Forward	AGGAGCCTGGCAACCATG
	Reverse	GATCCTCTGCCAGGAAACAG
IFI44L	Forward	GCTTCTAGCAGACATCAGAG
	Reverse	GAATGTCATCCATGCACAG
GAPDH	Forward	CTTTGGTATCGTGGAAGGACTC
	Reverse	GTAGAGGCAGGGATGATGTTC

Supplementary Table 4-18 and R script for WGCNA are deposited in figshare (https://figshare.com/articles/Supplementary_Table_4-14/11683959).

Supplementary Figure 1. Gating strategy

The gating strategy is shown. To evaluate $CD19^{+}$ B cells along two axes, $CD19^{+}$ B cells were first divided from peripheral blood mononuclear cells (A). Then, we defined subsets of B cells as follows: Bm1 cells; $CD38^{-}IgD^{+}$, naïve B cells; $CD38^{+}IgD^{+}$, pre-germinal centre (pre-GC) B cells; $CD38^{high}IgD^{+}$ and memory B cells; $CD38^{\pm}IgD^{-}$ (B).

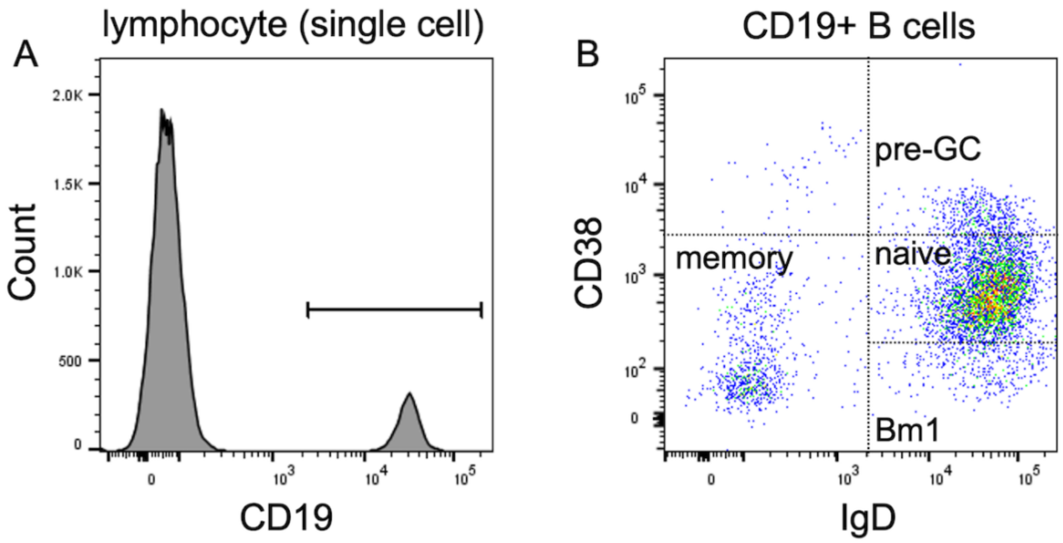

.

Supplementary Figure 2. Relative expression levels of *LINC00487* in B cell subsets. GCB, germinal centre B cell: HC, healthy controls: pSS, primary Sjögren's syndrome.

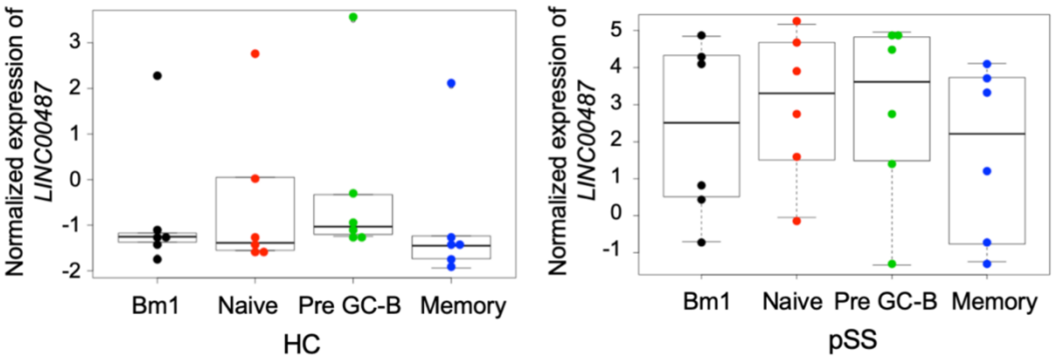

Supplementary Figure 3. Characteristics of *LINC00487*.

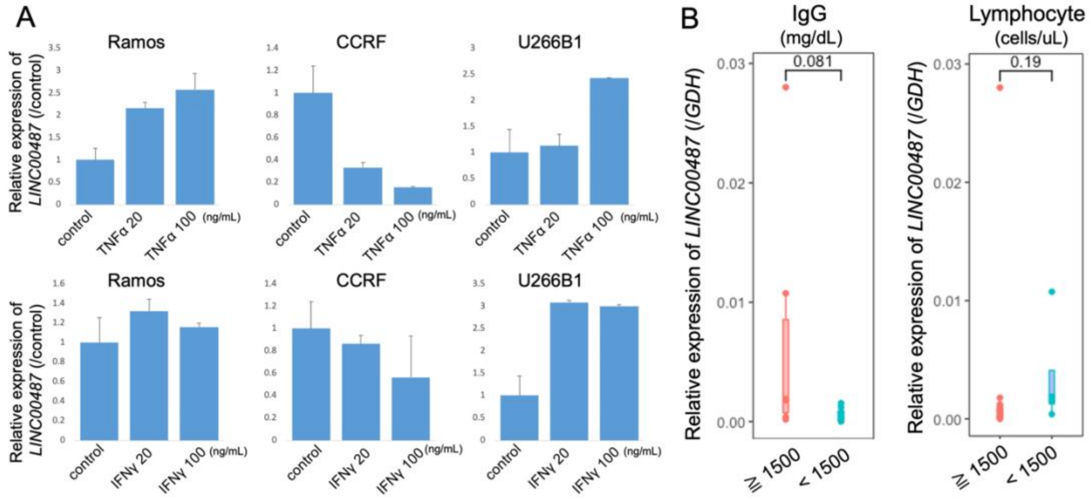

(A) qPCR analysis of *LINC00487* transcripts in the B cell lines after treatment with TNFα (above) and IFNγ (below). Cells were stimulated for 48 h. Results are represented as the mean ± standard deviation. (B) Comparison of the expression of *LINC00487* using qPCR in primary human CD19+ B cells derived from patients with pSS divided by serum IgG levels (left) and lymphocyte count (right) in the validation cohort. The P-value was calculated using the Mann–Whitney test.

Supplementary Figure 4. Weighted gene co-expression network analysis of B cell subsets of HCs.

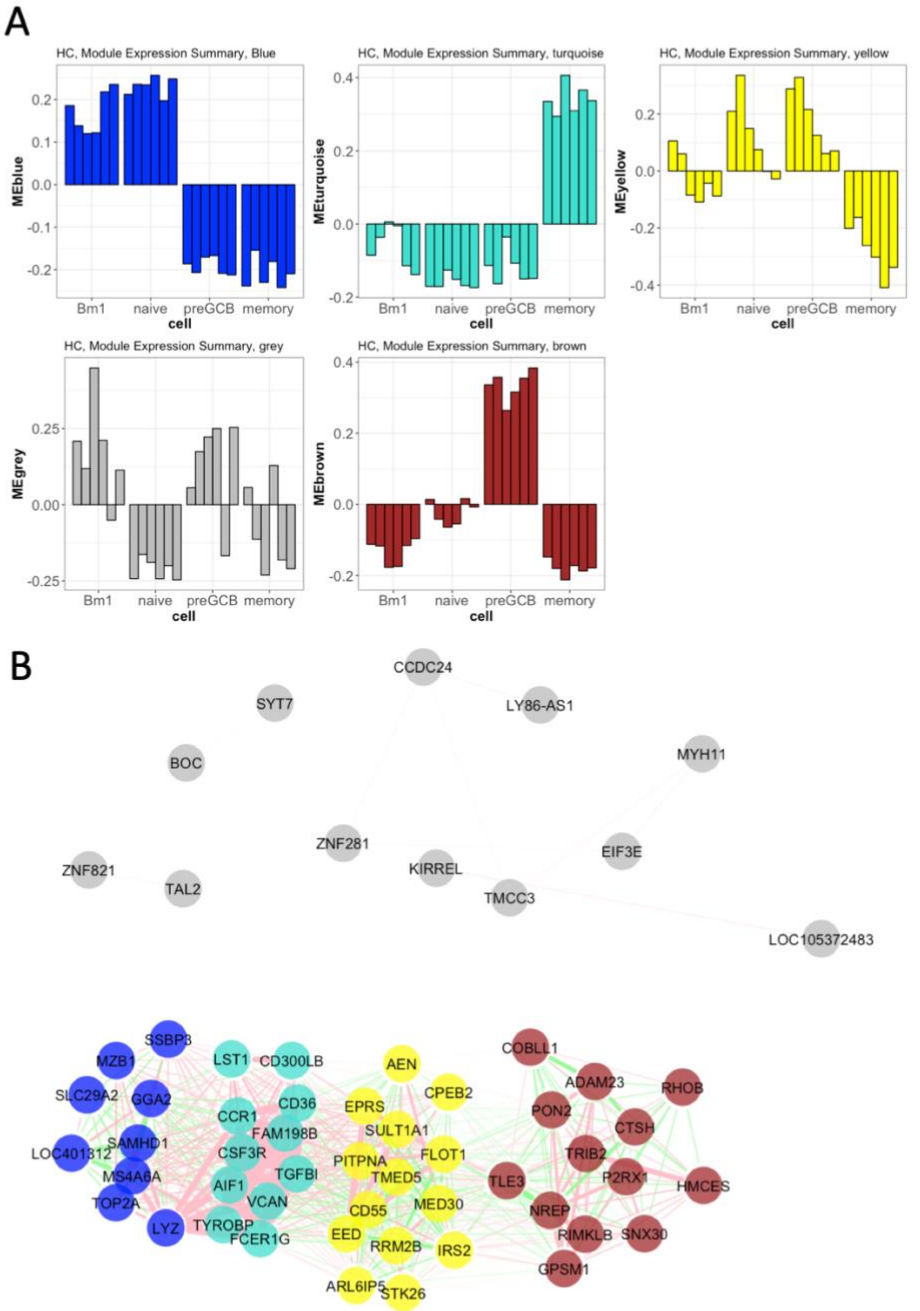

(A) Module expression in each B cell subset of HCs. The y-axis displays values of the module eigengene, and the x-axis displays each cell subset. (B) Associations of intra- and inter-modular hub genes in HCs. Node-colour corresponds to module-colour of HCs. The pink-coded edge represents the correlation, and the green-coded edge represents an inverse correlation. The width of the edge reflects the absolute weight of a correlation. GCB, germinal centre B cell.

Supplementary Figure 5. Venn diagram of genes in module.

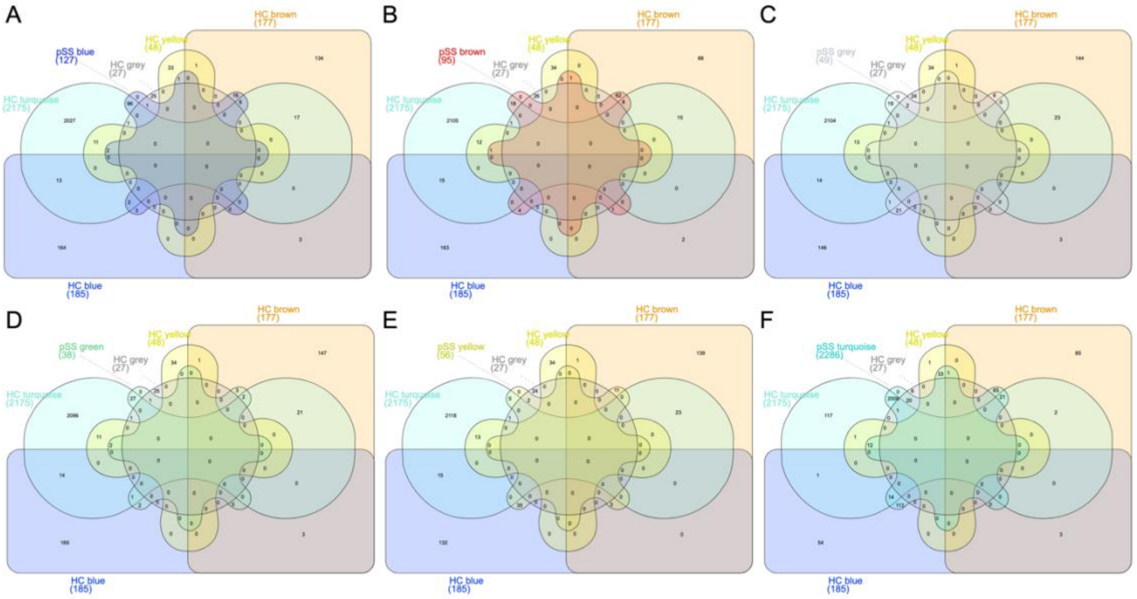

Venn diagram showing number of genes in pSS module of blue (A), brown (B), grey (C), green (D), yellow (E) and turquoise (F) overlapping with HC modules. HC, healthy controls; pSS, primary Sjögren's syndrome. Venn diagram was created using InteractiVenn [Heberle, H., et al. BMC Bioinformatics 16:169 (2015).].

Supplementary Figure 6. Top five significantly enriched canonical pathways (left), upstream regulators (middle) and disease/functions (right) specific for pSS. The colour of each frame corresponds to module-colour.

Pathway

yellow

Ingenuity Canonical Pathways	-log(p-value)
P2Y Purigenic Receptor Signaling Pathway	2.82
Role of NFAT in Cardiac Hypertrophy	2.66
FGF Signaling	2.64
Reelin Signaling in Neurons	2.60
Superpathway of Inositol Phosphate Compounds	2.59

turquoise

Ingenuity Canonical Pathways	-log(p-value)
p38 MAPK Signaling	1.56
Estrogen-mediated S-phase Entry	1.52
Th17 Activation Pathway	1.50
iNOS Signaling	1.48
Neuropathic Pain Signaling In Dorsal Horn Neurons	1.45

grey

Ingenuity Canonical Pathways	-log(p-value)
Cleavage and Polyadenylation of Pre-mRNA	1.53

brown

Ingenuity Canonical Pathways	-log(p-value)
Amyloid Processing	1.56
Ovarian Cancer Signaling	1.38
D-myo-inositol-5-phosphate Metabolism	1.34
Calcium Transport I	1.30

blue

Ingenuity Canonical Pathways	-log(p-value)
IL-17A Signaling in Airway Cells	2.76
Antiproliferative Role of Somatostatin Receptor 2	2.68
FcγRIIB Signaling in B Lymphocytes	2.62
AMPK Signaling	2.55
Role of p14/p19ARF in Tumor Suppression	2.55

green

Ingenuity Canonical Pathways	-log(p-value)
Diphthamide Biosynthesis	2.23
Trans, trans-farnesyl Diphosphate Biosynthesis	2.10
Mevalonate Pathway I	1.63
RAN Signaling	1.50
Superpathway of Geranylgeranyldiphosphate Biosynthesis I (via Mevalonate)	1.50

Upstream Regulator

yellow

Upstream Regulator	-log(p-value)
miR-21-5p	3.40
BRD1	3.00
miR-129-5p	2.71
WLS	2.38
pectin	2.21

turquoise

Upstream Regulator	-log(p-value)
SIM1	1.97
SREBF2	1.96
C3AR1	1.93
tamoxifen	1.93
LYZ	1.84

grey

Upstream Regulator	-log(p-value)
iberdomide	2.32
sapanisertib	1.85
myxothiazol	1.78
ATF5	1.78
HDAC10	1.72

brown

Upstream Regulator	-log(p-value)
miR-873-5p	3.57
mir-193	3.48
CTBP2	2.50
PTP4A3	2.45
isovaleric acid	2.28

blue

Upstream Regulator	-log(p-value)
BRD1	2.93
boldine	2.18
pectin	2.18
matrine	2.18
P4HA1	2.18

green

Upstream Regulator	-log(p-value)
NTNG2	2.71
miR-615-3p	2.24
HIC1	2.21
HOXC11	1.94
mir-503	1.87

Disease and bio functions

yellow

Disease and bio functions	-log(p-value)
Binding of phosphatidylinositol 3,5-diphosphate	3.97
Formation of artificial clathrin cages	3.95
Sebaceous gland tumor	3.86
Binding of phosphatidylinositol-3-phosphate	3.66
Binding of phosphatidylinositol 3,4-diphosphate	3.66

turquoise

Disease and bio functions	-log(p-value)
Degranulation of granulocytes	21.16
Cytolysis of lymphatic system cells	6.90
Cellular infiltration by blood cells	6.78
Response of liver	6.45
Aggressive non-Hodgkin lymphoma	6.18

grey

Disease and bio functions	-log(p-value)
Autosomal recessive congenital cataract	2.94
Lymphoid hyperplasia of small intestine	2.60
Dilated cardiomyopathy type 1T	2.60
Arrest in Gap 0-Gap 1 phase of embryonic cell lines	2.60
Arrest in Gap 0-Gap 1 phase of kidney cell lines	2.60

brown

Disease and bio functions	-log(p-value)
Morphology of vascular sprout	4.10
Papillary carcinoma of bladder	3.58
Arrest in cell cycle progression of breast cancer cell lines	3.26
Quantity of macropinosomes	3.13
Metastasis of cells	3.13

blue

Disease and bio functions	-log(p-value)
Targeting of cellular membrane	3.91
Infiltration by myofibroblasts	3.91
Abnormal morphology of pre-B lymphocytes	3.38
Formation of dendritic spines	3.28
Abnormal morphology of transitional B lymphocytes	3.22

green

Disease and bio functions	-log(p-value)
Synthesis of chondroitin sulfate	2.82
Entry into cell cycle progression of peripheral T lymphocyte	2.70
Neurodevelopmental disorder with dysmorphic facies and distal limb anomalies	2.70
Endocrine-cerebroosteodysplasia	2.70
Activation of CD4+ T-lymphocytes	2.65
